# Hospital Expenditure—The Commissariat

**Published:** 1897-03-13

**Authors:** 


### The Institutional Workshop.

### HOSPITAL EXPENDITURE—THE COMMISSARIAT.

### XXXV.—THE ANNUAL REPORT.

The sole medium of communication between the hospital and its subscribers is the annual report. The wards of the hospital are, of course, open to visits at reasonable times, but such cursory inspections, however interesting, cannot be very informing, and for all points relating to management and expenditure, even those who most warmly support and sympathise with the work are entirely dependent on the statements accompanying the yearly account and list of subscribers. The varied constitutions and methods of these institutions, which have sprung up, as it were, spontaneously all over the kingdom, and are subject to no law outside themselves, is nowhere more apparent than in the framing of these annual reports. Until quite recently no single basis of agreement outside the publication of the names of subscribers could be counted on in comparing one with another. The simplest method, and that which found most favour, was to convey as little information as was consistent with the appearance of imparting any at all. Often the report was modelled on some old form long since grown inadequate, but never adapted to suit a newer order of things. Sometimes, but rarely, much information of an ill-digested, and therefore almost useless, description, was set before the reader. In nearly every case all the emphasis was laid on raising money and the urgent need for more funds, while details relating to past expenditure were fragmentary and obscure. These defects might be attributed in great part to a dread on the part of the authorities of misconception arising if data for criticism were supplied to ignorant and irresponsible outsiders, and the alternate indifference and hostility of that large section of the public who never give a penny to help on hospital work lends a certain amount of justification to this attitude. But the slovenly form in which it was usual to present those items of information as to expenditure which could not be withheld, was due even more to want of system than to intentional obscurity. The Uniform System of Accounts, now generally adopted throughout the metropolis and in the leading provincial hospitals, has done immense service in clearing the ground from many time-honoured and complicated methods of "how not to do it," and has furnished manager and subscriber alike with a scheme so simple that the least skilled cannot blunder over its conclusions, and so comprehensive that no institution, however vast, can outrun its provisions. There are still places where the Uniform System of Accounts is unknown even by name, where soap reckons as an article of provisions (to give but one example), and where other similar absurdities baffle all attempts to gauge expenditure. But these may be left to time and public opinion.

It must be remembered that the Uniform System of Accounts is purely financial, and concerned exclusively with affording a clear presentment of moneys received and moneys spent. All the countless details which go to make up a good report outside the counting-house are foreign to its scope. Accordingly the amount of information conveyed as to the internal arrangements of the institution, the medical and surgical treatment of the patients, their nursing, and the domestic economy of the household, is left to the discretion of secretary and committee, with very varying results. Matters relating to the hospital commissariat, with which alone these articles are concerned, are almost without exception treated with the most conspicuous brevity. Taking into consideration the large amount of public attention which has been drawn of late years to the victualling of the hospitals, it is certainly remarkable that not one report which has hitherto come under the writer's notice supplies data for forming a just opinion on the domestic economy of the institution. For example: Let us suppose a subscriber to both Westminster and University Hospitals (which are of approximate size) to be taken with a desire to compare the reports he receives from both, in order to ascertain which is the more economically managed. He sees at a glance that in one hospital nearly £1,000 a year more is spent on provisions than in the other, and for this, search as he may, he can discover no explanation. What wonder if he forms an entirely erroneous impression, unassisted by the few figures which would have placed him in possession of the fact that in one hospital the whole staff of nurses is boarded under the general account, and in the other none. It is obvious to the commonest intelligence that the amount of the butcher's bill can never furnish any evidence as to economy unless at the same time the number boarded is mentioned.

As regards patients, it is possible to form a fair estimate as to number. It is usual now in all good reports to state not only the number of in-patients received in the year, but also whether they are medical or surgical cases, their average length of stay, and the daily average of occupied beds throughout the year. Without such supplementary information it is at once apparent that a statement of the number of in-patients received during the year may be absolutely misleading. Yet it is not uncommon in minor institutions to find merely this bare record, leaving it a matter of pure guess-work whether ten days or ten weeks' board has been supplied to each.

It would seem almost superfluous to emphasise the fact, if it were not so often left to the imagination, that the board of the whole staff and household is, with few exceptions, reckoned in hospitals with the board of patients. If, then, the public is to draw any conclusions at all relating to the economy of the hospital they are interested in supporting, the numbers boarded of the medical, administrative, and nursing staff, as well as of all servants and helpers, must be noted year by year with strict accuracy in the report. There can be no question of inviting criticism by laying bare the household management. This plea is now hopelessly out of date. The public will criticise, and the question is only whether they shall do it ignorantly and mischievously, or be provided with proper data for forming a sound opinion. As it stands now, certain figures only are available, and, unsupported by further information, they are fertile in promoting the most serious misconceptions, of far-reaching consequence for evil.

It may be objected, that even if the exact numbers boarded were set forth, there would still be many anomalies and discrepancies from which false deductions might be made. To this it may be added that any such discrepancies between one year and another, or any unusual rate of expenditure in special articles, might well be explained in notes to the accounts rendered. It is becoming usual to append the last year's accounts for purposes of comparison side by side with those of the current year. It might well be indicated in a footnote that the extra £100 on the milk bill was due to the introduction of milk for the nurses' lunch in lieu of beer, while a corresponding asterisk would point out the reduction in malt liquor. Or again, more scientific methods of making beef tea might account for a large reduction in the butcher's bill, and it would be to everyone's interest to point out this fact instead of leaving the reform a matter of mystery. As the numbers boarded in respective years would be clearly printed at the head of the accounts, any increase or decrease in patients, staff, or household would be instantly noted, and appear in its full significance.

To complete the value of these figures the annual cost of boarding each class of inmates might usefully be appended, with such other details at the disposition of the secretary as might prove of interest to kindred institutions, and be varied at discretion from year to year. It is rather sad to reflect how much valuable information on points relating to hospital finance is accumulated every year in the few really well-worked secretarial departments of our hospitals, and practically buried through some traditional distrust of publicity. Such information, digested and arranged in a form which could be readily grasped by all interested in the welfare of the hospitals, and presented year by year in well-selected variety in the annual report, would shed a flood of light into many dark corners, and lend just the helping hand to new or ill-managed institutions for which they may now seek everywhere in vain.

It is often said that none but those possessed of many years' experience of hospital management should venture into the field of hospital criticism. Let but those fully equipped for the campaign issue forth from the close ambush in which they are now hidden, and assuredly none will be found to dispute their right to labours exceptionally arduous and intricate, and affecting interests of paramount importance to the community at large.

